# Pulmonary influences on early post-operative recovery in patients after cytoreductive surgery and hyperthermic intraperitoneal chemotherapy treatment: a retrospective study

**DOI:** 10.1186/1477-7819-10-258

**Published:** 2012-11-27

**Authors:** Erebouni Arakelian, Michael R Torkzad, Antonina Bergman, Sten Rubertsson, Haile Mahteme

**Affiliations:** 1 Department of Surgical Sciences, Section of Surgery, Uppsala University, Uppsala, Sweden; 2 Department of Radiology, Oncology and Radiation Science, Section of Radiology, Uppsala University, Uppsala, Sweden; 3 Department of Surgical Sciences, Section of Anaesthesiology and Intensive Care, Uppsala University, Uppsala, Sweden

**Keywords:** Peritoneal carcinomatosis, CRS, HIPEC, Post-operative recovery, Pulmonary influences, Radiological assessment

## Abstract

**Background:**

The combination of cytoreductive surgery (CRS) and hyperthermic intraperitoneal chemotherapy (HIPEC) is a curative treatment option for peritoneal carcinomatosis (PC). There have been few studies on the pulmonary adverse events (AEs) affecting patient recovery after this treatment, thus this study investigated these factors.

**Methods:**

Between January 2005 and December 2006, clinical data on all pulmonary AEs and the recovery progress were reviewed for 76 patients with after CRS and HIPEC. Patients with pulmonary interventions (thoracocenthesis and chest tubes) were compared with the non-intervention patients. Two senior radiologists, blinded to the post-operative clinical course, separately graded the occurrence of pulmonary AEs.

**Results:**

Of the 76 patients, 6 had needed thoracocentesis and another 6 needed chest tubes. There were no differences in post-operative recovery between the intervention and non-intervention groups. The total number of days on mechanical ventilation, the length of stay in the intensive care unit, total length of hospital stay, tumor burden, and an American Society of Anesthesiologists (ASA) grade of greater than 2 were correlated with the occurrence of atelectasis and pleural effusion. Extensive atelectasis (grade 3 or higher) was seen in six patients, major pleural effusion (grade 3) in seven patients, and signs of heart failure (grade 1–2) in nine patients.

**Conclusions:**

Clinical and radiological post-operative pulmonary AEs are common after CRS and HIPEC. However, most of the pulmonary AEs did not affect post-operative recovery.

## Background

Peritoneal carcinomatosis (PC) can arise from various tissues, including the appendix, colorectal, abdominal mesothelioma and the ovaries. It is considered a terminal disease, and patients with this diagnosis usually have a poor prognosis despite treatment with systemic chemotherapy [1]. Cytoreductive surgery (CRS) combined with hyperthermic intraperitoneal chemotherapy (HIPEC) is a relatively recent treatment, and can be curative in selected patients with PC [1,2]. However, this treatment combination is a complex and time-consuming procedure. During CRS, tumor cells on the surface of intra-abdominal organs and the peritoneum are carbonized and destroyed with high voltage electrosurgical diathermy, which produces heat [3,4]. This results in a large intra-abdominal burn area, and may cause fluid loss. Furthermore, to avoid a critical rise in body temperature during HIPEC, systemic hypothermia is usually required before HIPEC [1,5-7]. CRS and HIPEC may result in hemodynamic changes as a result of moderate blood loss, peripheral vasodilatation, and massive fluid shift [5,7-9]. Thus, the patient’s general condition and their respiratory, cardiovascular, metabolic status, and electrolytic balance are significantly affected [8,9]. Large abdominal incisions, in addition to a long surgery and the particular operating technique used [3] may lead to a fluid deficit. Therefore, to maintain adequate circulating volume and urine output, large amounts of intravenous fluids are given during surgery [9-11].

As was reported previously, the initial post-operative care is recommended to take place in the intensive care unit (ICU) because of the hemodynamic and respiratory challenges for the patient after surgery, and the total hospital stay lasts about 3 weeks [12]. The post-operative recovery process may be influenced by several factors before, during and after the operation, and one of these factors is the incidence of pulmonary adverse events (AEs) [13]. However, there are few reports in the literature on pulmonary AEs occurring after CRS and HIPEC. Acute respiratory distress syndrome (ARDS)was reported in two case studies after CRS and HIPEC, with systemic inflammatory response or multiple transfusions during major surgery suggested as possible etiologies [10,14].

The primary aim of this study was to investigate the factors related to pulmonary AEs. In addition, we aimed to describe the effect of pulmonary AEs on the post-operative recovery process after CRS and HIPEC, and to compare patients who received an intervention with those who did not.

## Methods

### Ethics approval

The study was approved by the regional ethics committee, and was performed in accordance with the Declaration of Helsinki [15]. Informed consent was obtained from each patient.

### Patients

Between January 2005 and December 2006, 76 patients with PC (42 women) with a mean age of 54 years (range 24–75 years) and mean BMI of 25 (range 17–33) underwent primary CRS and HIPEC at Uppsala University Hospital, Uppsala, Sweden.

The inclusion criteria were: histologically confirmed diagnosis of PC; no distant metastasis; adequate renal, hematopoietic and liver functions and a WHO performance status of 3 or greater [16]. Table 1 demonstrates patient characteristics, from which the most of patients’ baseline data has been presented previously [12].

**Table 1:** **Patient characteristics**^a^

**Characteristics**	
Co-morbidity, n	
None	66
Asthma	1
Chronic obstructive lung disease	1
Cardiovascular diseases or diabetes	8
ASA: classification grade n	
1	36
2	33
3	3
Missing data	4
Diagnosis, n	
Pseudomyxoma peritonei	42
Colorectal adenocarcinoma	27
Ovarian cancer	6
Malignant abdominal mesothelioma	1
ICU stay, hours	37 [9–159]
Missing data, n	1
Total hospital stay, days	22 [11–56]

Abbreviations: ASA, American Association of Anesthesiologists.

^a^Some of the patients’ baseline data has been presented previously [12]. The PCwas extensive in the majority of patients, and therefore they underwent both upper and lower abdominal surgery. The American Society of Anesthesiologists physical status classification (ASA grade) was 1 or 2 in 91% of the patients, and only 2 of the 76 patients had pulmonary co-morbidities before surgery. The overall hospital stay was 3 weeks.

### Data collection

Data were collected on admission, progress, and discharge, operating room, ICU and post-operative ward stay, previous medical history, primary diagnosis, surgical procedures performed, tumor burden (Peritoneal Cancer Index; PCI), chemotherapy received before and after the operation, stoma formation, duration of surgery, ASA grade, peri-operative blood loss and fluid therapy, total time on mechanical ventilation, need for treatment with continuous positive airway pressure (CPAP), post-operative recovery progress, duration of stay in the ICU, and total hospital stay. All pulmonary AEs of grades II to V were documented in accordance with the Common Terminology Criteria (version 3.0) of the National Cancer Institute [13]. In addition, all available radiological images of the lungs taken during the first post-operative week were reviewed separately by two senior radiologists, who were blinded to all clinical data. The radiological imaging was performed when indicated by the clinical symptoms. Only two of the patients had any history of lung disease prior to surgery, and these had asthma and chronic obstructive pulmonary disease respectively.

### Anesthesia

All 74 patients had a thoracic epidural catheter inserted between the Th8 and Th10 level before induction of anesthesia. For medical reasons, two of the patients had patient-controlled analgesia instead of epidural anesthesia (EDA). Patients were given EDA (bupivacain and sufentanil) before surgery began, and this was continued up to 8 days postoperatively. Anesthesia was induced by fentanyl and sodium thiopental, and after administration of rocuronium and fentanyl, the trachea was intubated and mechanical ventilation started. Anasthesia was maintained with isoflurane and intermittent doses of fentanyl.

Large amounts of combined fluids (crystalloids and colloids) were administered peri-operatively, based on evaluation of the blood loss, blood pressure, pulse rate, central venous pressure, urinary output, body temperature, and skin turgor (Table 2; patient baseline data has also been presented previously [12]). Post-operative pain management was mainly based on epidural anesthesia but often other analgesics had to be added.

**Table 2:** **Peri-operative and post-operative fluid therapy**^a,b^

	**Mean (range)**	**Median**	**n (ND)**
Surgery			
Duration of surgery, hr: m	9:51 (3:20 to 16:40)	10:00	76
Ascites, ml	3,350 (150 to 11,000)	2,500	25(51)
Bleeding, ml	2,384 (50 to 14,000)	1,500	75 (1)
Total mechanical ventilation time, hr:m:s	00:22:52 (00:06:50 to 02:04:15)	00:09:45	76
CPAP, n			
Yes	15		
No	61		
Length of CPAP use, days	4.1 (1.0 to 10.0)	4.0	15
Fluids during surgery, ml			
Crystalloid	12,220(5,000 to 23,100)	11,750	76
Colloids	4,791 (550 to 21,150)	4,250	76
Sum of crystalloid and colloids	17,012 (5,900 to 35,150)	16,000	76
Intraoperative blood transfusion	1,130 (300 to 3,300)	900	56
Fresh frozen plasma	1,877300 to 8,400)	1,500	47
Total crystalloid balance, ml			
On the day of surgery	10,502 (1,455 to 20,820)	10,057	76
Post-op day 1	−77 (−3,982 to 4,498)	−182	66 (10)
Post-op day 2	−937 (−4,450 to 2,615)	−900	65 (11)
Post-op day 5	−405 (−3,800 to 3,277)	−450	69 (7)
From day of surgery until post-op day 5	7,292 (−4,790 to 22, 507)	7,025	76
Pre-op and post-op weight differences, kg			
Pre-operative	73.1 (45.3 to 110.0)	72.0	76
Post-op day 1	+4.6	+5.8	63 (13)
Post-op day 5	+0.7	+1.9	71 (5)
Before discharge	−3.9	−3.0	74 (2)

Abbreviations: CPAP, continuous positive airway pressure; ND, no data; op, operative.

^a^Some of the patients’ baseline data has been presented previously [12].

^b^Operating time was approximately 10 hours, and patients received some 17 litres of fluids during surgery.

^c^Balance was calculated as input (the amount fluids) given minus output.

^c^Post-operative weight increased on the first post-operative day after surgery, and decreased below baseline level before discharge.

### Cytoreductive surgery and hyperthermic intraperitoneal chemotherapy

CRS was performed as described by Sugarbaker, and HIPEC was given by means of the Coliseum technique [6,17]. The selection of drugs for HIPEC was based on previous studies [1] patients with PMP received mitomycin C and patients with colorectal cancer received oxaliplatin, while patients with ovarian cancer, gastric cancer, or mesothelioma were treated with a combination of cisplatinand doxorubicin. The duration of perfusion with oxaliplatin was 30 minutes, and for all other drugs it was 90 minutes. Tumor load and completeness of cytoreduction (CC) for peritoneal carcinomatosis were recorded using the PCI and the CC score directly after surgery, as described previously [1,17]. Of the 76 patients, 20 patients received pre-operative chemotherapy, and the mean PCIfor the patients was 23 (range 3–39, PCIwas missing for 13 patients). Diaphragm stripping was performed in 52 patients, and a stoma was created for 44 patients.

**Figure 1:**
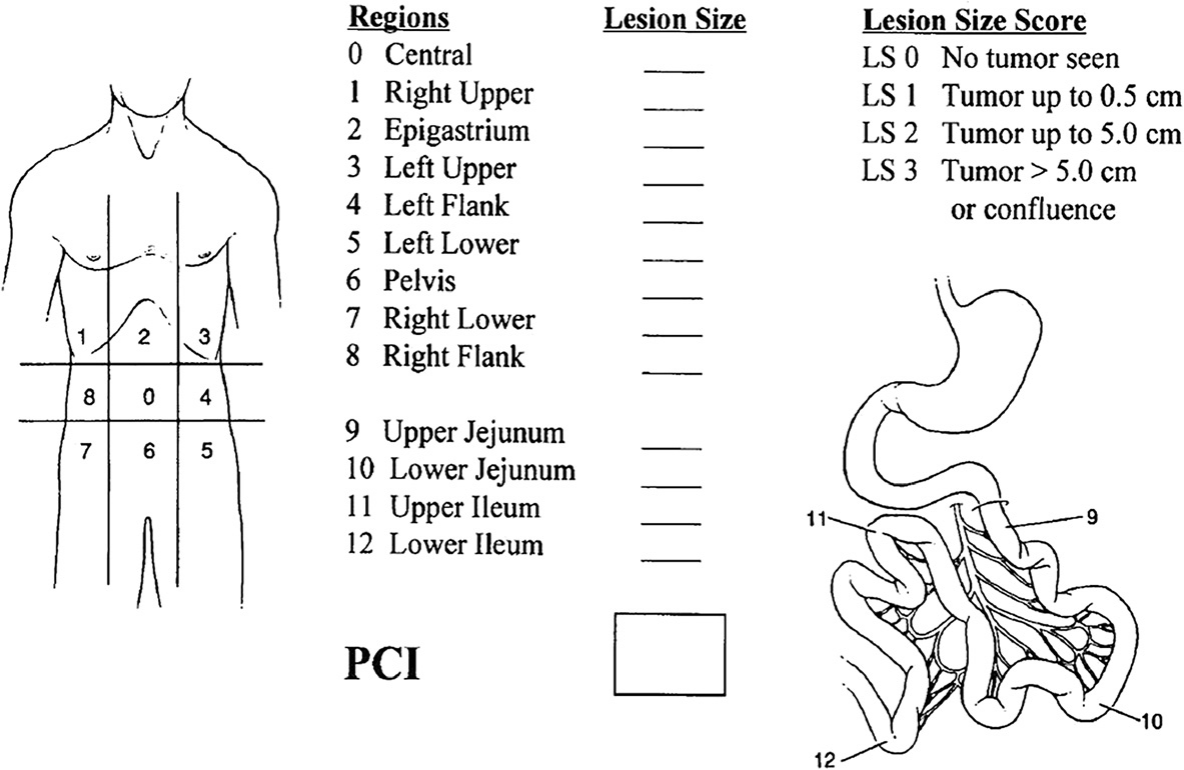
**Peritoneal Cancer Index (PCI).** PCI was used to measure the implant size and the distribution of the tumor in the abdomen. For the purpose of this study the abdomen was divided into: (**A**) upper abdominal regions (1 to 3), (**B**) middle abdominal regions (0, 4, 8 to12), and (**C**) lower abdominal regions 5 to 7. The PCI is printed with permission from the originator, Dr. Sugarbaker [18].

For the purpose of this study and to assess the pulmonary AEs resulting from the extent of the surgical trauma, the abdomen was divided with two transverse and two sagittal planes in a fashion similar to the abdominal-pelvic and small bowel regions of the PCI system [18] (Figure 1). All patients could then be classified as presented in Table 3. Thus, upper abdominal surgery only was performed for 6 patients, mid-abdominal only for 2, lower abdominal only for 16 patients, and both upper and lower abdominal for 52.

**Table 3:** Classifications of the operating site

**The operating site**	**Regions**	**Description**
Upper abdomen (n = 58)	1 to 3	Right upper quadrant, epigastrium, and left upper quadrant
Middle abdomen (n = 75)	0, 4, 8 to 12	Right flank, central, left flank, and small bowel
Lower abdomen (n = 68)	5 to 7	Right lower quadrant, pelvis, and left lower quadrant

^a^Regions were determined in accordance with the Peritoneal Cancer Index.

Upper abdominal surgery was performed for 58 patients, middle abdominal surgery for 75 patients, and lower abdominal surgery for 68 patients. Diaphragm stripping and resection of liver capsule was performed for 52 patients.

### Postoperative recovery

Post-operative recovery of patients in this study was defined as mobilization [19] (patients standing up, sitting down on a chair, washing themselves, and walking for the first time after surgery), and drinking, eating, and having bowel movements or flatulence [19]. Patients were mobilized in accordance with a specially designed mobilization schedule after surgery [12].

### Radiological assessment of the thoracic organs and grading of pulmonary adverse events

The radiological images included chest radiography and thoracic computed tomography (CT). For the purposes of this study, gradations were assessed on opacities or infiltrates in each lung consistent with atelectasis, pleural effusion on each side, and signs of congestive heart failure (CHF). The gradations are presented in Table 4. For patients who had images taken more than once during the first post-operative week, the image rated as the higher grade was used.

**Table 4:** **Gradation of pulmonary adverse events made for the purpose of this study**^**a**^

**Pulmonary AE/Grade**	**Description**
Atelectasis	
0	No opacity
1	Lamellar
2	Segmental
3	Lobar
4	More extensive than one lobe
Pleural effusion	
0	None present
1	Minimal amounts defined as blunted pleural sinus
2	Moderate amounts defined as extension to two pleural sinuses but not reaching the level of lung hilum
3	Large amounts up to and > the level of lung hilum
Signs indicative of heart failure	
0	No signs of heart failure
1	Enlargement of pulmonary vessels in the absence of other congestive signs (pulmonary effusion and/or cardiac enlargement) was interpreted as suggestive of congestion
2	Congestive heart failure was diagnosed when dilatation or congestion of pulmonary vessels were present, combined with pleural effusion and/or cardiac enlargement

^a^Atelectasis was graded based on the extension of the opacity, pleural effusion was graded by the amount, and signs indicative of heart failure were noted collectively.

### The intervention group

With respect to progress in their recovery process, patients who had an invasive intervention as a result of their pulmonary AE(s) were compared with those who received no intervention. Invasive intervention (thoracocentesis and chest tubes) was performed if there was presence of respiratory distress, including dyspnea, tachypnea, or poor saturation.

### Statistical analysis

Inter-rater analysis between the two radiologists’ gradations of atelectasis, pleural effusion, and heart failure was performed using Cohen’s *κ-*value. To test the probability of association between the effects of pulmonary AE on recovery variables, the Mann–Whitney *U*-test or Kruskal–Wallis test was performed. To test the probability of association between clinical data on post-operative recovery variables, univariate analysis of each clinical and post-operative recovery variables was tested using the *χ*^2^ or Fishers’ exact test for categorical data, and the Mann–Whitney *U*-test or Kruskal–Wallis test for continuous data. A general linear model in a multivariate analysis was used for correlation analysis between post-operative recovery variables and pulmonary AEs with a 95% confidence interval (CI). Correlation analysis between different variables was tested using Spearman’s rank correlation. *P <* 0.05 was considered significant.

## Results

### General considerations

Sixty-two patients had post-operative imaging, 60 of which were chest radiographs. Both atelectasis and pleural effusion in grades 1–4 were seen in 69% of the patients, and 15% had enlargement, dilatation or congestion of pulmonary vessels (Table 5). Cohen’s weighted kappa (κ) value between the two radiologists was 0.45 for atelectasis and 0.41 for pleural effusion and heart failure, indicating moderate agreement between the two radiologists.

**Table 5:** Pulmonary adverse events occurring in 62 patients (60 radiographs and 2 computed tomography scans) during post-surgery week 1, and comparison between gradations performed by radiologist 1 and 2

	**Radiologist 1**	**Radiologist 2**	**Cohen’s weighted κ value**
Atelectasis (n)			
None (grade 0)	20	14	
Lamellar atelectasis (grade 1)	13	15	0.45
Segmental atelectasis (grade 2)	23	8	
Lobar and larger than lobar (≥ grade 3)	6	25	
Pleural effusion (n)			
None (grade 0)	20	11	0.41
Minimal (grade 1)	12	18	
Moderate (grade 2)	23	19	
Large (grade 3)	7	14	
CHF (n)			
None	53	48	
Signs suggestive of CHF (grade 1)	6	8	0.41
CHF (grade 2^a^)	3	6	
Pneumonia	2		
Empyema	1		
Respiratory insufficiency	1		

Abbreviations: CHF, congestive heart failure.

^a^Moderate heart failure.

Six patients developed extensive atelectasis (≥ grade 3). Major pleural effusion (grade 3) was observed in seven patients and signs of heart failure (grade 1–2) developed in nine patients. Two patients developed pneumonia, one had empyema, and another patient developed respiratory insufficiency. Cohen’s weighted value κ, indicated a moderate level of agreement between the two radiologists.

The presence of atelectasis, pleural effusion, and heart failure showed no correlation with gender, age (<65 vs. ≥65 years), BMI, different types of primary tumor, duration of surgery, stoma formation, pre-operative chemotherapy, presence of AEs (surgical, infectious, or medical); type of surgical procedure (upper, middle, or lower abdominal) or presence of diaphragm stripping. Pulmonary AEs were not correlated with mobilization (as defined in the Methods section).

### Factors influencing the occurrence of atelectasis and pleural effusion

There was a correlation between the occurrence of atelectasis and total mechanical ventilation time (Table 6). Moreover, it was found that patients with segmental or larger atelectasis (grade 2 or higher) were extubated a mean of 1.2 days later than those with atelectasis grades 0 to 1. A correlation was found between the length of ICU stay or total hospital stay and the occurrence of atelectasis.

**Table 6:** Effect of pulmonary adverse events (AEs) on recovery time and parameters of62 patients

**Recovery**^a^	**Pulmonary AE**				***R *value**	***P-*value**
	**Type**	**Grade**	**Mean**	**95% CI**		
MV time, days	Atelectasis	0 to 1	2.1	1.2 to 3.1	0.35	0.02
		2 to 3	3.3	2.4 to 4.2		
ICU stay, days	Atelectasis	0 to 1	2.0	1.4 to 2.5	0.38	0.03
		2 to 3	1.1	0.9 to 1.3		
Total hospital stay, days	Atelectasis	0 to 1	21.1	18.0 to 24.2	0.11	0.02
		2 to 3	22.8	20.3 to 25.3		
ICU stay, days	Pulmonary effusion	0	1.0	0.9 to 1.2	0.33	0.02
		2	2.0	1.3 to 2.6		
PCI	Pulmonary effusion	0	19	13 to 24	0.95	0.02
ASA		1 to 2	30	23 to 36	0.28	0.02

Abbreviations ASA, American Society of Anesthesiologists; ICU, intensive care unit; MV, mechanical ventilation; PCI, Peritoneal Cancer Index.

^a^Total mechanical ventilation, length of ICU stay, total hospital stay, tumor burden, and ASA correlated with the occurrence of atelectasis and pleural effusion.

In a multivariate analysis, pleural effusion correlated with PCI and ASA classification grade. Patients with pleural effusion of grades 1 to 2had higher PCI (>24) than those who had no pleural effusion, and there was a correlation between ASA classification grade and pleural effusion. The duration of stay in the ICU also correlated with pleural effusion, and a *post hoc* test showed that patients with a moderate amount of pleural effusion (grade 2) had a mean stay of approximately 23 hourslongerin the ICU than the patients with no effusion.

The occurrence of heart failure in nine patients correlated with oral intake (*r* 0.33, *P* 0.02), bowel movement (*r* 0.26, *P* 0.03), length of ICU stay (*r* 0.38, *P* 0.03) and length of total hospital stay (*r* 0.30, *P* 0.02).

### Early post-operative recovery and volume of peri-operative fluids

Patients received a mean of approximately 17 liters of combined crystalloids and colloids during surgery, which resulted in a positive fluid balance on the day of surgery. No correlations were found between the peri-operative fluid therapy used (crystalloids, colloids, and the sum of both) and the occurrence of post-operative pulmonary AEs or whether upper or lower abdominal surgery had been performed. Patients’ pre-operative fluid balance decreased gradually during the 5 days after surgery, while weight, increased on post-operative day 1, but decreased before discharge to below the baseline level (*P* < 0.005).

### Recovery comparison between pulmonary intervention and non-intervention groups

In total, 12 patients needed an invasive intervention to treat pulmonary AEs; 6 patients underwent thoracocentesis and 6 others received chest tubes. None of the patients was re-intubated as a result of a pulmonary AE. For patients in the intervention group, eating, drinking, and bowel functions were restored within a mean of 14 days, and it took a mean of 6 days for patients to stand up, sit down on a chair, wash themselves, and walk independently after the surgery.

The mobilization process, restoration of gastrointestinal functions and patient characteristics were the same for the patients in the intervention group as for those in the non-intervention group. There was a difference between the intervention and non-intervention groups with regard to the amount of peri-operative crystalloids (*P* 0.02) and the total amount of crystalloids and colloids (*P* 0.02) needed. Compared with patients in the non-intervention group, patients in the intervention group received approximately 3 litres more of crystalloids and 4 liters more of combined crystalloids and colloids. In the intervention group, 4 patients needed CPAP compared with 11 patients in the non-intervention group, and the mean duration of CPAP was approximately 4 days longer in the intervention group than in the non-intervention group (*P* 0.02) (Table 7).

**Table 7:** **Comparisons in recovery process of the 12 patients in the intervention group with 64 non-intervention patients**^a^

**Peri-operative and post-operative parameters**	**Intervention group, mean (95% CI)**	**Non-intervention group, mean (95% CI)**	***P*-value**^**b**^
Use of CPAP days	6.8 (3.2 to 10.3)	3.1(1.4 to 4.8)	0.02
Peri-operative crystalloids, ml	14,842 (12,100 to 17,582)	11,671 (10,677 to 12,665)	0.02
Sum of peri-operativecrystalloids and colloids, ml	20,629 (16,790 to 24,469)	16,255 (14,702 to 17,808)	0.02

CPAP, continuous positive airway pressure.

^a^In total, 12 patients needed an invasive intervention due to their pulmonary adverse events. The recovery process, restoration of gastrointestinal functions, the length of ICU stay and total hospital stay were the same as for the patients in the non-intervention group. Use of CPAP was longer for the patients in the intervention group and this group also received larger amounts of peri-operative crystalloids and combined crystalloids and colloids

^b^All *P*-values were significant.

## Discussion

### Pulmonary complications after cytoreductive surgery and hyperthermic intraperitoneal chemotherapy

In this study, we found that pulmonary AEs are common after CRS and HIPEC; however, despite this, only a limited number of patients (16%) needed thoracocentesis or chest tubes. This may be a result of the strict patient selection (good performance status) for this treatment [1], as most of the study patients (91%) were ASA grade 1 or 2, and only two patients with pulmonary AEs had co-morbidities prior to surgery.

Patients after CRS and HIPEC treatment, usually required early post-operative ICU treatment due to influence on thoracic organs. However, the extent of treatment influence on thoracic organs is unknown. In the literature, pulmonary AEs after CRS and HIPEC have been reported previously in only two case reports, which both described the occurrence of ARDS after CRS and HIPEC [10,14].

### Peri-operative fluids and other clinical factors

To study the effect of peri-operative trauma and its effects on thoracic organs, we divided the abdomen into three surgical sites: upper, middle, and lower abdomen. We considered that the surgical trauma and the surgical site closest to the diaphragm (upper abdomen), the large amount of fluids administered during surgery, and the length of time that patients were kept on a respirator might have an influence on the development of pulmonary AEs [20,21]. However, despite stripping of the diaphragm, the patients included in this study did not have any of the factors associated with pulmonary AEs. Therefore, insertion of a chest tube after surgery on the diaphragm should be based on individual patient signs and symptoms of AEs rather than the procedure.

Formation of atelectasis may be affected by several factors, including type and duration of anesthesia, patient position, inhaled fraction of oxygen [20], lack of positive end-expiratory pressure, and presence of paralysis caused by muscle relaxants [20,22]. One study also showed that extubation failure may occur due to generous fluid treatment [23,24]. Conversely, we did not find that the amount of fluid therapy received correlated with the occurrence of atelectasis, pleural effusion, or heart failure. However, the patients who had thoracocentesis or who had chest tubes implanted received larger amounts of crystalloids and the combination of crystalloids and colloids during surgery. None of the study patients had any signs of heart failure prior to surgery, and most of the study patients (70%) developed no signs of CHFpostoperatively. In this study, there was a weak correlation between oral intake, bowel movement, and ICU stay with the occurrence of heart failure grade 1 or 2. However, because of small number of patients with post-operative signs of congestion or CHF, it is not possible to draw any reliable conclusions.

### Post-operative recovery

Pulmonary AEs were not correlated with recovery parameters (restoring gastrointestinal functions and mobilization). Nevertheless, it seems that atelectasis might influence the length of post-operative hospitalization and ICU stay. Although all the patients (from our previous cohort [12]) were supposed to follow the same post-operative mobilization schedule [12] regardless of the grade of their pulmonary AE, it is possible that patients with larger atelectasis had more extensive physical therapy and mobilization than the other patients and thus stayed in the ICU for a shorter time period than patients with less atelectasis.

In our study, patients with segmental and larger atelectasis were extubated later than other groups, and patients with moderate pleural effusion had the longest hospital stay, a finding that has also been suggested in earlier studies [20]. Conversely, good pain relief can result in patients being easier to mobilize and this might thereby result in smaller or less atelectasis. In this study, the number of patients with severe atelectasis may have been underestimated, and therefore having a larger number of patients with consecutive CT scans would have been preferable to allow us study the causality closely.

Patients in the intervention group, received larger amounts of crystalloids and the combination of colloids and crystalloids during surgery than the non-intervention group. A weight increase was seen in the entire patient population on post-operative day 1. Nevertheless, our centre’s policy is to give restricted fluid therapy during HIPEC, which is in line with other studies [6,8,11]. Despite good performance status, patients with higher PCI were more likely to develop pleural effusion, and this might reflect the extent of surgery. However, in this study there were no significant differences in PCI between the intervention and the non-intervention group. The intervention group also did not differ from the non-intervention group with respect to the restoration of gastrointestinal functions and mobilization.

### Issues concerning radiological imaging

Radiological imaging was not routinely carried out in this study, but was performed whenever the patients status required it, for example in cases of pulmonary AE. Images were taken of only 82% of the patients, and therefore some information about the pulmonary AEs may be lacking. However, this study reflects the real-life situation, because it describes the daily care of patients with PC at our hospital. Most of the images were bedside chest radiographs, and only two CT scans were performed. It is difficult to compare the two types of image because CT scans may show more detailed findings than chest radiographs [20,25,26], and this could influence the findings in this study. In the future, it would be preferable to perform radiologic examinations (such as ultrasonography, CT, and chest radiography) on predetermined dates in order to be able to draw better conclusions about pulmonary AEs after CRS and HIPEC. The benefits of these two imaging methods for patients undergoing major surgery could not be addressed in the current study.

This study, assessed the incidence of pulmonary AEs after CRS and HIPEC and their effect on early recovery. Although the gradations of atelectasis, pleural effusion and heart failure have not been established, we found similar results in the literature [20,27]. However, the radiologists who performed the gradations were blinded to the post-operative course of the study, and Cohen’s weighted κ score indicated only a moderate level of agreement between the two radiologists, which is a weakness in the study, but still demonstrates that the grading process was rigorous.

## Conclusion

In conclusion, clinical and radiological post-operative pulmonary AEs are common after CRS and HIPEC; however, most have no effect on post-operative recovery.

## Abbreviations

AEs: Adverse events; ARDS: Acute Respiratory Distress Syndrome; ASA: American Society of Anaesthesiologists; BMI: Body Mass Index; CC: Completeness of cytoreduction; CHF: Congestive heart failure; CI: Confidence interval; CPAP: Continuous positive airway pressure; CRS: Cytoreductive surgery; CT: Computed tomography; EDA: Epidural anesthesia; HIPEC: Hyperthermic intraperitoneal chemotherapy; ICU: Intensive care unit; PC: Peritoneal carcinomatosis; PCI: Peritoneal Cancer Index.

## Competing interests

The authors declare that they have no competing interests.

## Authors’ contributions

EA designed and conducted the study, analyzed the data, and helped to write the manuscript. MT helped to design the study, conducted the radiological assessments, and helped to write the manuscript. AB conducted the radiological assessments and helped to write the manuscript. SR helped to design the study and helped to write the manuscript. HM is the principal investigator, and designed the study, assisted in writing, revising and editing the manuscript. All authors approved the final manuscript.
